# Distribution of Virulence Genes in *Campylobacter* spp. Isolated from *Agaricus* Mushrooms in Iran

**DOI:** 10.1155/2023/1872655

**Published:** 2023-01-31

**Authors:** Maryam Sadat Emami, Amir Shakerian, Reza Sherafati Chaleshtori, Ebrahim Rahimi

**Affiliations:** ^1^Department of Food Hygiene, Faculty of Veterinary Medicine, Shahrekord Branch, Islamic Azad University, Shahrekord, Iran; ^2^Research Center of Nutrition and Organic Products, Shahrekord Branch, Islamic Azad University, Shahrekord, Iran; ^3^Research Center for Biochemistry and Nutrition in Metabolic Disease, Kashan University of Medical Sciences, Kashan, Iran

## Abstract

The white button mushroom (*Agaricus*) is a significant nutritional and therapeutic species utilized in the human diet and could transmit various bacterial infections. *Campylobacter* species are the most common cause of foodborne illness across the world. The present study has been planned to determine the frequency of virulence genes and antibiotic susceptibility test in *Campylobacter* spp. recovered from *Agaricus* mushroom. In this study, 740 *Agaricus* mushroom samples were gathered randomly from various markets from June 2020 to December 2020. Confirmation of *Campylobacter* spp. using biochemical analyses and *23S rRNA*-based PCR was performed. The agar dilution technique was used to determine resistance to antibiotics using gentamicin (GM10*μ*g), ciprofloxacin (CIP5*μ*g), nalidixic acid (NA30*μ*g), tetracycline (TE30*μ*g), ampicillin (AM10*μ*g), amoxicillin+ clavulanic acid (AMC30*μ*g), erythromycine (E15*μ*g), azithromycin (AZM15*μ*g), clindamycin (CC2*μ*g), and chloramphenicol (C30*μ*g). Multiplex PCR was utilized to determine the prevalence of the *recR*, *dnaJ*, *wlaN*, *virBll*, *cdtC*, *cdtB*, *cdtA*, *flaA*, *cadF*, *pidA*, *ciaB*, *ceuE*, and *cgtB* genes. *Campylobacter* spp. were detected in 74 out of 740 *Agaricus* mushroom samples (10%). According to the data, *Agaricus* mushroom samples included 32 (4.32%) *C. jejuni*, 11 (1.48%) *C. coli*, and 31 (4.18%) other *Campylobacter* spp. Antimicrobial resistance was most common in *C. jejuni* isolates. *C. jejuni* isolates also had the lowest resistance rate to gentamycin, ciprofloxacin, and nalidixic acid. *C. coli* isolates were reported to have the highest antimicrobial resistance to ciprofloxacin, ampicillin, and erythromycine. Resistance to gentamycin and amoxicillin+ clavulanic acid was likewise lowest among *C. coli* strains. The *flaA* and *ciaB* genes were found in 100% of B-lactams-susceptible *C. jejuni* and *C. coli* strains. When examining the relationship between antibiotic resistance and the existence of virulence genes, it was observed that there is a statistically significant relationship (*p* < 0.001) between bacterial resistance and virulence genes. Our findings indicated that changes in resistance patterns in *Campylobacter* strains have emerged from multiple treatment approaches in *Agaricus* mushrooms.

## 1. Introduction

Mushrooms have been utilized in food and medical supplies for decades and have become a vital element of human nutrition. Due to their high-quality nutrients, carbohydrates, enzymes, essential fatty acids, dietary fibers, and low-calorie content, these substances have an appealing taste, fragrance, and nutritive quality [1]. Their production and sales have increased dramatically in recent years all over the globe. Mushrooms are regarded as a regular diet and a supplier of nutritional supplements because of their favorable impact on humans due to their presence of bioactive components and nutritional ingredients [2]. The white button (*Agaricus*) mushroom is among the essential edible mushrooms that have long been sought by people searching for food. The Chinese recognized the advantages of mushrooms, believing that mushrooms enhance the physical organs and prolong vitality and vigor [3]. This mushroom is being utilized as a diet and medicinal plant in Iran and other nations. *Agaricus* mushrooms are particularly fragile because they lack a defensive cuticle, have a rapid breathing function, and contain much wetness. Consequently, they are subjected to mechanical degradation, pathogen assault, weight loss, and caramelization, resulting in a fast-postharvest reduction in quality. *Agaricus* mushrooms have a storage life of one to three days at room temperature (20–25°C) and five to seven days under a refrigerator (4°C) [4].

Mushrooms are ideal hosts for a wide range of pathogens. Various microorganisms grow on the surface of mushrooms that might infect humans. One of the most significant bacteria is *Campylobacter* [5]. *Campylobacter* spp., a primary cause of bacteremia gastroenteritis in mammals, are frequently found in animal digestive tracts and, through fecal pollution, in animal-derived products. Raw milk, chickens, and beef have all been related epidemiologically to epidemics of *Campylobacter* infection in humans [6, 7]. *Campylobacter* can be identified in 1 to 1.5% of agricultural packaged food. As a result, several items that are epidemiologically linked to *Campylobacter* outbreaks have been recognized as responsible for the transmission of the *Campylobacter* genera [8]. The Seattle-King Health Center researched to quantify the migration of *Salmonella* spp. and *Campylobacter* spp. from animals to humans through the food supply chain and discovered that people with *Campylobacter* enteritis ate mushrooms [9]. As a result, there seemed to be an increased relative incidence of *Campylobacter* infection in humans in people who consumed mushrooms. Our goal was to see if raw mushrooms are a reservoir of *Campylobacter* spp. [10]. Freshly *Agaricus* mushroom packets sold at local supermarket shops are high in *Campylobacter* [11].

*Campylobacter* can cause bladder infections, pneumonia, or neuropathies, including septic arthritis, Guillain-Barre syndrome (GBS), inflammatory bowel disease, and Miller-Fisher syndrome (MFS). The Agaricus mushroom population causes approximately 40% of people's problems. Recent genetic investigations have shed insight on the primary virulence components involved in the *Campylobacter* strain's virulence. Furthermore, the capacity of *Campylobacter* to attach via the *cadF*, *racR*, *virB11*, *pldA*, and dnaJ proteins infiltrates intestinal mucosa cells by the *ciaB* and *ceuE* genes and manufactures toxins via the *cdtA*, *cdtB*, and *cdtC* genes [12]. Even though *Campylobacter* diseases are typically self-limiting and do not require treatment, antimicrobial therapy is not necessary in most cases of protracted disease in humans and septicemia. Also, it is essential in some types of sepsis. The selection medications for treating human *campylobacter*iosis include macrolides (erythromycin and azithromycin), fluoroquinolones (ciprofloxacin), and tetracyclines. The primary reasons for rising susceptibility patterns are the overuse of antibiotics in human illnesses and abusing antibacterial medications in animal farming to treat animal diseases or boost growth by adding antibiotics to food within the *Campylobacter* genus [13]. Antimicrobial resistance is caused by several biological processes that have been well-documented. The chloroquine inhibition domain of the DNA gyrase gene, *gyrA*, is primarily responsible for fluoroquinolone resistance. The acquired *tet(O)* gene, which codes for a defensive ribosomal polypeptide, is usually linked to high tetracycline resistance. Mutations in the V motif of the *23S rRNA* gene and stimulation of the *CmeABC* multidrug efflux system are commonly linked to antibiotic resistance to macrolides. Antibiotic-resistant genes like *erm(B)*, *aadE*, or *sat4* (streptomycin/streptothricin resistance), *blaOXA-61* (b-lactams resistance), and *aphA-3* (aminoglycosides resistance) have also been linked to multidrug resistance in *Campylobacter* isolates [14]. Numerous studies have found a relationship between virulence and antibiotic susceptibility in bacterial infections, implying a correlation between antibiotic resistance and the bacteria's ability to colonize or invasion. This link has been investigated, and some studies have found that infection with antibiotic-resistant *Campylobacter* isolates in people is linked to a prolonged length of diarrhea [15, 16]. In this research, we focused on the incidence of *Campylobacter* infection in *Agaricus* mushrooms in Iran and the infection distribution and antimicrobial resistance in collected isolates. As a result, this analysis is aimed at looking into the genetics of antimicrobial resistance (AMR) and finding virulence indicators in *Campylobacter* spp. samples from *Agaricus* mushrooms.

## 2. Methods

### 2.1. Biochemical Identification of *Campylobacter* spp.

In this investigation, 740 *Agaricus* mushroom samples were gathered randomly from various markets from June 2020 to December 2020 and transferred to the laboratory. The selection represented the two varieties of *Agaricus* mushroom brands produced in Iran. Samples were placed below 4°C during transportation, and testing was performed immediately after receiving the samples. When the *Agaricus* mushroom trends turned up in the lab, they were torn into pieces and blended in a sterile folder with a sterilized swab stick, and roughly 1 g of each template was homogenized in enrichment broth and incubated for 24 hours at 37°C. Homogenized solution was then scrubbed onto (predried) *Campylobacter* agar base (Sigma-Aldrich, Germany) plates supplemented with Karmali selective complement SR0167E (Sigma-Aldrich, Germany). Samples were incubated for 48 hours at 42°C in an anaerobic jar with a gas-generating sachet (Oxoid–CampyGen™) to establish a microaerophilic environment for the growth of *Campylobacter* spp.

On 24–48 h Karmali agar plates, colonies with a clean, flat, colorless, transparent to the grey appearance of diameters of 1 mm were chosen. Since colonies are frequently mixed with other microorganisms, the mobility of suspected bacteria was examined using a section contrast microscope. Colonies exhibiting exceptional mobility were isolated on 5% horse blood agar plates and incubated for 48 hours in an anaerobic jar with a fuel-producing sachet to generate microaerophilic conditions. Gram-negative, catalase-positive, and oxidase-positive samples were snap-frozen in glycerol broth at -70°C for further molecular characterization (PCR), antibiotic sensitivity testing, and resistance gene identification [17].

### 2.2. Confirmation of *Campylobacter* spp. Using *23S rRNA*-Based PCR

#### 2.2.1. DNA Extraction

In an anaerobic jar with a gas-generating pouch, frozen *Campylobacter* isolates were grown on 5% horse blood agar plates and incubated for 48 hours at 42°C. The bacterial cells were taken from the plates and put into Eppendorf vials containing 200 *μ*L of sterile water when they had grown sufficiently. The suspensions were heated for 8 minutes at 98°C on a boiling tube. The supernatant was collected and was then placed into sterile microcentrifuge tubes and centrifuged at 17000 g for 5 minutes to serve as a genetic DNA material for the following polymerase chain reaction (PCR). The quality (A260/A280) and amount of the extracted DNA were next measured at an optical density of 260/280 nm using a spectrophotometer (NanoDrop, Thermo Scientific, Waltham, MA, USA). The DNA's validity was tested on a 1.5% agarose gel stained with ethidium bromide (0.5 g/mL) (Thermo Fisher Scientific, St. Leon-Rot, Germany). The polymerase chain reaction (PCR) was carried out using a PCR thermal cycler (Eppendorf Co., Hamburg, Germany) according to the Tohid and Shandiz technique [18].

#### 2.2.2. Molecular Identification of *Campylobacter* Species

Corroborating the identity established using biochemical techniques, primers encoding the *Campylobacter* genus-specific *23S rRNA* gene and species-specific sequences of *Campylobacter jejuni*, *Campylobacter coli*, and other *Campylobacter* strains were utilized (Table 1). Five microliters of 5x PCR buffer, 4 mmol^−1^ MgCl2, 2 *μ*L of 2 mmol^−1^ deoxynucleotide triphosphate (dNTPs), 0.5 *μ*L of 25 pmol each of oligonucleotide primers, and 1 U of *Taq* polymerase (Promega) with 2 *μ*L DNA template were included in the precursor solution. The volume was adjusted to 25 *μ*L using deionized water. PCR conditions for *23S rRNA* gene detection were 30 cycles of denaturation at 95°C for 30 seconds; annealing at 46°C temperatures for 30 seconds, elongation at 72°C for 30 seconds, and final extension at 72°C for 7 minutes were used in the amplification. Electrophoresis with a 1.5% agarose gel including ethidium bromide was used to examine the PCR results [17, 18].

### 2.3. Antibiotic Resistance Analysis

The Kirby-Bauer procedure was used for antimicrobial disk susceptibility test. The Mueller-Hinton agar (Zist. Rouyesh, Tehran, Iran) plates containing 5% defibrinated sheep blood as a medium was used to evaluate antibacterial sensitivities to various antibacterial drugs in a twofold serial dilution tend to range from 0.063 to 128 *μ*g/mL^−1^ depending on the Medical and Laboratory Standard rules (M100) [17]. The inocula were made by swabbing two to three colonies from 24 h culture into a sterile 0.85% sodium chloride (NaCl) solution to create a cell suspension that matched the 0.5 McFarland threshold. This seed contained about 2 × 10^8^ CFU mL^−1^, which was then adjusted 1 : 10 to achieve a ratio of 10^7^ CFU mL^−1^. This was injected onto multiple plates holding varied antimicrobial doses using a multipoint inoculator. To allow the *Campylobacter* spp. to proliferate, the cultures were kept at 42°C for 24 hours in an anaerobic jar with a gas-generating pouch (Oxoid–CampyGen™). The following antibiotics were used in this study: gentamicin (GM10*μ*g), ciprofloxacin (CIP5*μ*g), nalidixic acid (NA30*μ*g), tetracycline (TE30*μ*g), ampicillin (AM10*μ*g), amoxicillin+ clavulanic acid (AMC30*μ*g), erythromycine (E15*μ*g), azithromycin (AZM15*μ*g), clindamycin (CC2*μ*g), and chloramphenicol (C30*μ*g) [19].

### 2.4. Virulence Encoding Gene Detection

Multiplex PCR was used to determine the prevalence of the *recR*, *dnaJ*, *wlaN*, *virbll*, *cdtC*, *cdtB*, *cdtA*, *flaA*, *cadF*, *pidA*, *ciaB*, *ceuE*, and *cgtB* genes [20–23]. The primers and PCR conditions used to genotype the *recR*, *dnaJ*, *wlaN*, *virbll*, *cdtC*, *cdtB*, *cdtA*, *flaA*, *cadF*, *pidA*, *ciaB*, *ceuE*, and *cgtB* alleles are listed in Table 1. This amplified procedure was used in a multiplex PCR: 5 minutes of initial denaturation at 94°C, followed by 30 cycles of denaturation at 94°C for 30 seconds, annealing at 54°C for 30 seconds, and elongation at 72°C for 1 minute. In a final reaction volume of 25 *μ*L, each solution contained 4 mmol^−1^ MgCl2, 1 *μ*L of 25 pmol per primer, 2 *μ*L of 2 mmol^−1^ dNTPs, and 4 *μ*L of 5x PCR-buffer, as well as 1 U of *Taq* polymerase (Promega) and 2 *μ*L DNA template. After that, the PCR electrophoresis was performed on a 1.5% agarose gel with ethidium bromide in a 1x TBE buffer. By measuring the sizes of the individual amplicons to a 100 bp ladder, the lengths of the different amplicons were established.

### 2.5. Statistical Analysis

The frequency of virulence genes was compared among species of bacteria (*C. jejuni* vs. *C. coli*) using a multivariate analysis of variance (ANOVA) with the number of genes as the dependent variable and the *Campylobacter* species as the factor analyzed with SPSS statistical software (version 24). When the *p* value was less than 0.05, the results appeared significant.

## 3. Results

### 3.1. Frequency of *Campylobacter* spp.

The prevalence of *Campylobacter* spp. was investigated in 740 *Agaricus* mushroom specimens. To quickly identify *Campylobacter* spp., the Gram-staining, catalase, and oxidase analyses were utilized. After incubation, the 74 positive *Campylobacter* spp. were recognized by catalase and oxidase tests, which produced a purple hue, a blue/purple tint, and the formation of oxygen bubbles, respectively. *Campylobacter* spp. were detected in 74 out of 740 *Agaricus* mushroom samples (10%). According to the data, *Agaricus* mushroom samples included 32 (4.32%) *C. jejuni*, 11 (1.48%) *C. coli*, and 31 (4.18%) other *Campylobacter* spp.

All of the organisms were confirmed using PCR amplification of the *23S rRNA* gene. All 74 isolates were positive for *Campylobacter* spp. according to PCR data. *C. jejuni* (4.32%) and *C. coli* (1.48%) had the highest prevalence of *Campylobacter* spp. bacteria, whereas other *Campylobacter* spp. were detected in 31 samples (5.33%). There was a significant statistical difference (*p* < 0.05) between the samples and the prevalence of *Campylobacter* infections. In this study, the results demonstrated that biochemical test identification accuracy was not significantly different from molecular PCR test accuracy (*p* < 0.05).

### 3.2. Antibiotic Susceptibility Test of *Campylobacter* Isolates

Antimicrobial resistance profiles of *Campylobacter* isolates were recovered from multiple specimens (Tables 2 and 3). *C. jejuni* isolates exhibited high resistance against tetracycline, ampicillin, amoxicillin+ clavulanic acid, and erythromycine. On the other hand, *C. jejuni* isolates had the lowest rate of resistance to gentamycin, ciprofloxacin, and nalidixic acid. Furthermore, a significant percentage of resistance to antibiotics azithromycin (40.62%), clindamycin (25%), and chloramphenicol (37.5%) was observed in different isolates. There was a statistical difference between the specimens and antimicrobial resistance incidence (*p* < 0.05).

*C. coli* isolates were reported to have the highest antimicrobial resistance against ciprofloxacin (72.72%), ampicillin (72.72%), and erythromycin (72.72%). However, the resistance to gentamycin (0%) and amoxicillin+ clavulanic acid (27.27%) was noticed to be lowest among *C. coli* strains. The results, on the other hand, revealed that both *C. coli* and *C. jejuni* isolates were completely sensitive to antibiotics gentamycin.

### 3.3. Prevalence of Virulence Factors

The rates of virulence genes among resistant isolates of *C. jejuni* were as follows for *recR*, *dnaJ*, *cdtC*, *cdtB*, *cdtA*, *flaA*, *cadF*, and *ciaB*, respectively. The *flaA* and *ciaB* genes were found in 100% (32/32) of *C. jejuni* strains when these genes were tested in susceptible isolates. The frequency of these genes in *C. jejuni* was noticed to be the lowest when the *wlaN*, *virbll*, and *ceuE* genes were examined (Table 3).

Table 3 shows the genotype distribution of *C. coli* isolates collected from various types of specimens. *FlaA* and *ciaB* were the most prevalent genotypes observed among *C. coli* recovered from mushroom (100%). *DnaJ*, *wlaN*, *virbll*, and *ceuE* were the *C. coli* strains found with the lowest frequency in samples (0%). The *recR*, *cdtC*, *cdtB*, *cdtA*, *cgtB*, *cadF*, and *pidA* genes were also discovered in a variety of *C. coli* isolates. These genes were found in between ten and forty percent of the population. There was a significant difference (*p* < 0.05) between the types of samples and the occurrence of genotypes.

### 3.4. Association of Virulence Genes with the Antibiotic Resistance Pattern

**Figure 1:**
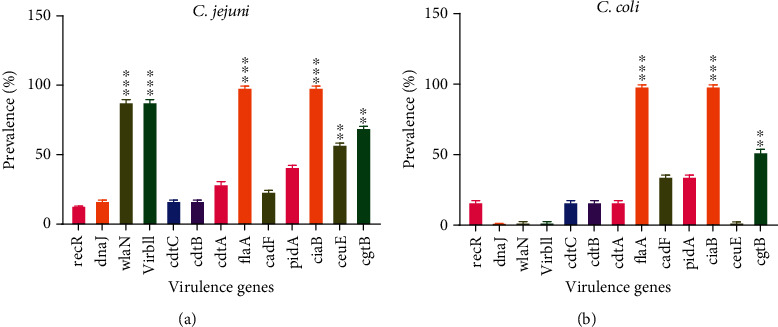
The prevalence of virulence genes in susceptible *C. jejuni* (a) and *C. coli* (b) isolates. Legend: virulence genes (*recR*, *dnaJ*, *wlaN*, *virbll*, *cdtC*, *cdtB*, *cdtA*, *flaA*, *cadF*, *pidA*, *ciaB*, *ceuE*, and *cgtB*). When researchers looked at the association between resistance to antibiotics and the presence of genetic variants, they discovered a positive significant association (^∗∗∗^*p* < 0.001) among bacterial resistance and the presence of virulence genes.

In terms of the association between the existence of virulence genes and antibiotic susceptibility/resistance profiles across strains, resistant *C. jejuni* isolates had more virulence-related genes than sensitive ones. Tetracycline-resistant isolates had more virulence genes than nalidixic acid and gentamycin-resistant strains isolated. The antibiotics GM10 and the *virBll* and *wlaN* genes, which exhibit no variability (100% resistance), were omitted from the study. When examining the relationship between antibiotic resistance and the existence of virulence genes in *C. jejuni* isolates, it was shown that there is a statistically significant relationship (*p* < 0.001) between bacterial resistance and the existence of virulence genes (Figure 1(a)). The medicines GM10 were excluded out of the *C. coli* investigation, as were the *dnaJ*, *virbll*, *ceuE*, and *wlaN* genes, which had no variability (100% resistance). When researchers looked at the association between resistance to antibiotics and the presence of genetic variants in *C. coli* strains, they discovered a positive significant association (*p* < 0.001) among bacterial resistance and the presence of virulence genes (Figure 1(b)).

## 4. Discussion

*Campylobacter* bacteria cause foodborne infections, and MDR variants are a severe health problem. There has been no information on pathogenic molecular features of regional *Campylobacter* isolates because there have been few investigations on the bacteria in Iran [24]. As a result, the presence of virulence in *Campylobacter* genera isolated from *Agaricus* mushrooms was investigated in this work. Several researchers worldwide have found that gene expression related to mobility, colonization, epithelium invasion, and toxin generation is crucial in the development of *Campylobacter*-related illnesses [25–28]. Most of the strains in this investigation were found to have associated virulence genes linked to pathogenic adherence, colonization, and invasive features. This was in line with prior research, which had found *flaA*, *ciaB*, *racR*, *virB11*, and *pldA* to be often prevalent [23].

Furthermore, as described by various investigations [29, 30], the *cdtA*, *cdtB*, and *cdtC* alleles required to produce the CDT toxic substance were found in all *Campylobacter* strains. Concerning *C. jejuni*, 59% of the examined strains had the *ceuE* gene, which confers the ability to chelate iron. The *wlaN* gene was found in 90% of *Campylobacter* strains, consistent with research conducted in Iran [31], which found a high incidence of isolated *Campylobacter* strains (82.22%). Furthermore, the *cgtB* gene was found in 54% of *C. coli* and 71% of *C. jejuni*. As a result, we believe that the high prevalence of these alleles among the tested samples might imply their significant pathogenic capability and high danger to human health. Lipooligosaccharide of *Campylobacter* (LOS), similar to gangliosides in neurons, is considered a critical factor in the initiation of GBS neuropathies and Miller-Fisher syndrome after *C. jejuni* infection [31]. The higher prevalence of these genes may be associated with GBS in humans. Antibiotic resistance, a global concern for animal and human health, has received much attention. Because of the extensive utilization of antibiotics in the food sector, antibiotic resistance has become a significant problem [32]. Previous research has found that *Campylobacter* has a high antimicrobial resistance to various drugs [33–40]. Antimicrobial resistance patterns correspond well with the presence of genes expressing resistance to antibiotics, according to a study examining the genetic basis of antibiotic resistance in isolates tested [34, 35].

Our findings demonstrated that multiple virulence factors are related to resistant bacteria when examining the frequency of virulence genes and antibiotic susceptibility. Indeed, the detection of *cadF* and *ciaB* in amoxicillin/clavulanic acid-resistant bacteria, *ciaB* in ampicillin-resistant bacteria, *racR* in nalidixic acid-resistant isolates, and *cadF* and *ceuE* in chloramphenicol-resistant isolates was linked. Although the presence of positive or negative relationships among antibiotic resistance and virulence genes in microorganisms has been demonstrated, the *Campylobacter* species remains contentious [41–43]. *In vitro* experiments have shown that resistant bacteria invade more than susceptible strains, whereas others have highlighted the tendency of susceptible strains to produce more serious diseases than resistant organisms. In the current investigation, virulence genes and antibiotic resistance among *C. jejuni* samples were shown to have some favorable relationships (*p* > 0.05). A few virulence genes linked to antimicrobial-resistant *C. jejuni* isolates are implicated in bacterial attachment and invasion, indicating that resistant strains have more adherence and attack potential than susceptible strains. Further research is needed to understand the association between virulent characteristics and antibiotic resistance in greater depth in *Campylobacter* isolates.

## 5. Conclusions

In the present study, a significant recovered rate of *Campylobacter* was found. Antibiotic resistance in *Campylobacter* strains isolated from *Agaricus* mushroom is a growing source of worry and might pose a severe public health danger. Resistance to multiple antibiotics with a significant association with virulent factors has been discovered. *Agaricus* mushroom *Campylobacter* strains from Iran have little resistance to antibiotics important to global health.

## Figures and Tables

**Table 1:** Primers of antibiotic resistance genes, annealing temperatures, and size of amplicons.

Gene	Primer sequences (5′-3′)	Annealing temperatures (°C)	Product size (bp)
*23S rRNA*	*23S rRNA*: TTAGCTAATGTTGCCCGTACCG ERY2075: TAGTAAAGGTCCACGGGGTCGC	46	485
*recR*	F: GATGATCCTGACTTTG R: TCTCCTATTTTTACCC	49	584
*dnaJ*	F: AAGGCTTTGGCTCATC R: CTTTTTGTTCATCGTT	53	720
*wlaN*	F: TTAAGAGCAAGATATGAAGGTG R: CCATTTGAATTGATATTTTTG	46	672
*virbll*	F: TCTTGTGAGTTGCCTTACCCCTTTT R: CCTGCGTGTCCTGTGTTATTTACCC	48	494
*cdtC*	F: CGATGAGTTAAAACAAAAAGATA R: TTGGCATTATAGAAAATACAGTT	47	182
*cdtB*	F: CAGAAAGCAAATGGAGTGTT R: AGCTAAAAGCGGTGGAGTAT	51	620
*cdtA*	F: CCTTGTGATGCAAGCAATC R: ACACTCCATTTGCTTTCTG	55	370
*flaA*	F: AATAAAAATGCTCATAAAAACAGGTG R: TACCGAACCAATGTCTGCTCTGATT	55	855
*cadF*	F: TTACTCCTACACCGTAGT R: AAACTATGCTAACGCTGGTT	45	283
*pidA*	F: AAGCTTATGCGTTTTT R: TATAAGGCTTTCTCCA	45	913
*ciaB*	F: TGGGCAGATGTGGATAGAGCTTGGA R: TAGTGCTGGTCGTCCCACATAAAG	44	284
*ceuE*	F: CCTGCTACGGTGAAAGTTTTGC R: GATCTTTTTGTTTTGTGCTGC	48	793
*cgtB*	F: TAAGAGCAAGATATGAAGGTG R: GCACATAGAGAACGCTACAA	50	561

**Table 2:** Antibiotic resistance pattern of *C. jejuni* and *C. coli* isolates recovered from *Agaricus* mushroom.

	Antibiotics	*GM10*	*CIP5*	*NA30*	*TE30*	*AM10*	*AMC30*	*E15*	*AZM15*	*CC2*	*C30*
*C. jejuni*	Number and the percentages of the isolates	2 (6.25%)	6 (18.75%)	6 (18.75%)	15 (46.87%)	16 (50%)	13 (40.62%)	17 (53.12%)	13 (40.62%)	8 (25%)	12 (37.5%)
*C. coli*		0 (0%)	8 (72.72%)	5 (45.45%)	4 (36.36%)	8 (72.72%)	3 (27.27%)	8 (72.72%)	4 (36.36%)	5 (45.45%)	5 (45.45%)

Legend: gentamicin (GM10*μ*g), ciprofloxacin (CIP5*μ*g), nalidixic acid (NA30*μ*g), tetracycline (TE30*μ*g), ampicillin (AM10*μ*g), amoxicillin+ clavulanic acid (AMC30*μ*g), erythromycine (E15*μ*g), azithromycin (AZM15*μ*g), clindamycin (CC2*μ*g), and chloramphenicol (C30*μ*g).

**Table 3:** Prevalence of virulence factors in *C. jejuni and C. coli*.

	*recR*	*dnaJ*	*wlaN*	*virbll*	*cdtC*	*cdtB*	*cdtA*	*flaA*	*cadF*	*pidA*	*ciaB*	*ceuE*	*cgtB*
*C. jejuni*	27 (84.37%)	26 (81.25%)	3 (9.37%)	3 (9.37%)	26 (81.25)	26 (81.25%)	22 (68.75%)	31 (96.87%)	24 (75%)	18 (56.25%)	32 (100%)	13 (40.62%)	9 (28.12%)
*C. coli*	2 (18.18%)	0 (0%)	0 (0%)	0 (0%)	2 (18.18%)	2 (18.18%)	2 (18.18%)	11 (100%)	7 (63.63%)	7 (63.63%)	11 (100%)	0 (0%)	11 (45.45%)

Legend: virulence genes (*recR*, *dnaJ*, *wlaN*, *virbll*, *cdtC*, *cdtB*, *cdtA*, *flaA*, *cadF*, *pidA*, *ciaB*, *ceuE*, and *cgtB*).

## Data Availability

The datasets used and analyzed during the current study are available from the corresponding author upon reasonable request.

## References

[1] Valverde M. E., Hernández-Pérez T., Paredes-López O. (2015). Edible mushrooms: improving human health and promoting quality life. *International Journal of Microbiology*.

[2] Fernandes T., Garrine C., Ferrão J., Bell V., Varzakas T. (2021). Mushroom nutrition as preventative healthcare in sub-Saharan Africa. *Applied Sciences*.

[3] Asad F., Anwar H., Yassine H. M., Ullah M. I., Kamran Z., Sohail M. U. (2020). White button mushroom, *Agaricus* bisporus (Agaricomycetes), and a probiotics mixture supplementation correct dyslipidemia without influencing the colon microbiome profile in hypercholesterolemic rats. *International Journal of Medicinal Mushrooms*.

[4] Hyde K. D., Xu J., Rapior S. (2019). The amazing potential of fungi: 50 ways we can exploit fungi industrially. *Fungal Diversity*.

[5] Shakerian A., Rokni N. D., Sharifzadeh A., Alagha S., Talebian R. (2005). Campylobacter jejuni as a potential pathogen in liver of broilers chickens in slaughtered & retail market broilers in Shahrekord, Iran. *Iranian Journal of Food Science and Technology*.

[6] Piri‐Gharaghie T., Beiranvand S., Riahi A. (2022). Fabrication and characterization of thymol-loaded chitosan nanogels: improved antibacterial and anti-biofilm activities with negligible cytotoxicity. *Chemistry & Biodiversity*.

[7] Abd El-Hamid M. I., Abd El-Aziz N. K., Samir M. (2019). Genetic diversity of Campylobacter jejuni isolated from avian and human sources in Egypt. *Frontiers in Microbiology*.

[8] Thomas K. M., de Glanville W. A., Barker G. C. (2020). Prevalence of *Campylobacter* and Salmonella in African food animals and meat: a systematic review and meta-analysis. *International Journal of Food Microbiology*.

[9] Piri Gharaghie T., Hajimohammadi S. (2021). Comparison of anti-candida effects of aqueous, ethanolic extracts and essential oil of E. angustifolia with fluconazole on the growth of clinical strains of Candida. *New Cellular and Molecular Biotechnology Journal*.

[10] Hoffmann S., Ashton L., Todd J. E., Ahn J. W., Berck P. (2021). *Attributing U.S. Campylobacteriosis cases to food sources, season, and temperature*.

[11] Jiang H., Miraglia D., Ranucci D. (2018). High microbial loads found in minimally-processed sliced mushrooms from Italian market. *Italian Journal of Food Safety*.

[12] Pavlovic I., Caro Petrović V., Bojkovski J. Gastrointestinal helminths of sheep breed in spread Belgrade area in period 2018-2019.

[13] Shafiei A., Rahimi E., Shakerian A. (2020). Prevalence, virulence and anti-microbial resistance in campylobacter spp. from routine slaughtered ruminants, as a concern of public health (case: Chaharmahal and Bakhtiari Province, Iran). *Research*.

[14] Shakerian A. (2016). Campylobacter spp. as a potential pathogen in the edible mushrum (Agaricus mushrooms). *Journal of Food Microbiology*.

[15] Sabzmeydani A., Rahimi E., Shakerian A. (2020). Incidence and antimicrobial resistance of campylobacter species isolated from poultry eggshell samples. *Egyptian Journal of Veterinary Sciences*.

[16] Sabzmeydani A., Rahimi E., Shakerian A. (2020). Incidence and antibiotic resistance properties of Campylobacter species isolated from poultry meat. *International Journal of Enteric Pathogens*.

[17] Panzenhagen P., Portes A. B., Dos Santos A. M., Duque S. D., Conte Junior C. A. (2021). The distribution of *Campylobacter jejuni* virulence genes in genomes worldwide derived from the NCBI pathogen detection database. *Genes*.

[18] Tohid G. P., Shandiz S. A. S. (2018). The inhibitory effects of silver nanoparticles on bap gene expression in antibiotic-resistant Acientobacter bumanni isolates using real-time PCR. *Journal of Ilam University of Medical Sciences*.

[19] Szczepanska B., Andrzejewska M., Spica D., Klawe J. J. (2017). Prevalence and antimicrobial resistance of *Campylobacter* jejuni and *Campylobacter* coli isolated from children and environmental sources in urban and suburban areas. *BMC Microbiology*.

[20] Gharbi M., Béjaoui A., Hamda C. B., Ghedira K., Ghram A., Maaroufi A. (2022). Distribution of virulence and antibiotic resistance genes in *Campylobacter jejuni* and *Campylobacter coli* isolated from broiler chickens in Tunisia. *Journal of Microbiology, Immunology and Infection*.

[21] Hennequin C., Robin F. (2016). Correlation between antimicrobial resistance and virulence in Klebsiella pneumoniae. *European Journal of Clinical Microbiology & Infectious Diseases*.

[22] Bardoň J., Pudova V., Koláčková I., Karpíšková R., Röderová M., Kolář M. (2017). Virulence and antibiotic resistance genes in *Campylobacter spp.* in the Czech Republic. *Epidemiologie, mikrobiologie, imunologie: casopis Spolecnosti pro epidemiologii a mikrobiologii Ceske lekarske spolecnosti JE Purkyne.*.

[23] Levican A., Ramos-Tapia I., Briceño I. (2019). Genomic analysis of Chilean strains of *Campylobacter* jejuni from human faeces. *BioMed Research International*.

[24] Rahimi E., Shakerian A., Kazemeini H. R., Goudarzi M. A. (2013). Antimicrobial resistance patterns of Campylobacter spp. isolated from raw chicken, Turkey, quail, partridge, ostrich, beef, sheep, goat and camel meat marketed in Shahrekord. *Journal of Food Technology and Nutrition*.

[25] Liu F., Ma R., Wang Y., Zhang L. (2018). The clinical importance of *Campylobacter* concisus and other human hosted *Campylobacter* species. *Frontiers in Cellular and Infection Microbiology*.

[26] Cayrou C., Barratt N. A., Ketley J. M., Bayliss C. D. (2021). Phase variation during host colonization and invasion by *Campylobacter* jejuni and other *Campylobacter* species. *Frontiers in Microbiology*.

[27] Bronowski C., James C. E., Winstanley C. (2014). Role of environmental survival in transmission of *Campylobacter* jejuni. *FEMS Microbiology Letters*.

[28] Ammar A. M., Abd M. I., El-Hamid R. M. S. (2021). Molecular detection of fluoroquinolone resistance among multidrug-, extensively drug-, and pan-drug-resistant Campylobacter species in Egypt. *Antibiotics*.

[29] Wysok B., Wojtacka J., Kivistö R. (2020). Pathogenicity of *Campylobacter* strains of poultry and human origin from Poland. *International Journal of Food Microbiology*.

[30] Sierra-Arguello Y. M., Perdoncini G., Rodrigues L. B. (2021). Identification of pathogenic genes in *Campylobacter* jejuni isolated from broiler carcasses and broiler slaughterhouses. *Scientific Reports*.

[31] Casabonne C., Gonzalez A., Aquili V., Subils T., Balague C. (2016). Prevalence of seven virulence genes of *Campylobacter jejuni* isolated from patients with diarrhea in Rosario, Argentina. *International Journal of Infection*.

[32] Khoshbakht R., Tabatabaei M., Hosseinzadeh S., Shekarforoush S. S., Aski H. S. (2013). Distribution of nine virulence-associated genes in *Campylobacter* jejuni and *C. coli* isolated from broiler feces in Shiraz, Southern Iran. *Foodborne Pathogens and Disease*.

[33] Piri Gharaghie T., Sadat Shandiz S. A., Beiranvand S. (2020). Evaluation of silver nanoparticles effects on bla-per1 gene expression for biofilm formation in isolates of antibiotic-resistant Acientobacter bumanni by real time PCR method. *Cellular and Molecular Researches (Iranian Journal of Biology)*.

[34] Farzi N., Yadegar A., Sadeghi A. (2019). High prevalence of antibiotic resistance in Iranian Helicobacter pylori isolates: importance of functional and mutational analysis of resistance genes and virulence genotyping. *Journal of Clinical Medicine*.

[35] Schiaffino F., Colston J. M., Paredes-Olortegui M. (2019). Antibiotic resistance ofCampylobacterSpecies in a pediatric cohort study. *Antimicrobial Agents and Chemotherapy*.

[36] Marotta F., Garofolo G., Di Marcantonio L. (2019). Antimicrobial resistance genotypes and phenotypes of *Campylobacter* jejuni isolated in Italy from humans, birds from wild and urban habitats, and poultry. *PLoS One*.

[37] Maksimović Z., Dizdarević J., Babić S., Rifatbegović M. (2022). Antimicrobial resistance in coagulase-positive staphylococci isolated from various animals in Bosnia and Herzegovina. *Microbial Drug Resistance*.

[38] Feldgarden M., Brover V., Haft D. H. (2019). Validating the AMRFinder tool and resistance gene database by using antimicrobial resistance genotype-phenotype correlations in a collection of isolates [published correction appears in Antimicrob Agents Chemother. 2020 Mar 24;64(4):]. *Antimicrobial Agents and Chemotherapy*.

[39] Aljazzar A., Abd M. I., El-Hamid R. M. S. (2022). Prevalence and antimicrobial susceptibility of campylobacter species with particular focus on the growth promoting, immunostimulant and anti-Campylobacter jejuni activities of eugenol and trans-cinnamaldehyde mixture in broiler chickens. *Animals*.

[40] El-Naenaeey E.-s. Y., Abd El-Hamid M. I., Khalifa E. K. (2021). Prevalence and antibiotic resistance patterns of Campylobacter species isolated from different sources in Eygpt. *Journal of Microbiology, Biotechnology and Food Sciences*.

[41] Duarte A., Santos A., Manageiro V. (2014). Human, food and animal *Campylobacter spp.* isolated in Portugal: high genetic diversity and antibiotic resistance rates. *International Journal of Antimicrobial Agents.*.

[42] Ammar A. M., El-Naenaeey E.-S. Y., El-Malt R. M. S. (2021). Prevalence, antimicrobial susceptibility, virulence and genotyping of Campylobacter jejuni with a special reference to the anti-virulence potential of eugenol and beta-resorcylic acid on some multi-drug resistant isolates in Egypt. *Animals*.

[43] Aleksić E., Miljković-Selimović B., Tambur Z., Aleksić N., Biočanin V., Avramov S. (2021). Resistance to antibiotics in thermophilic *Campylobacter*s. *Frontiers in Medicine*.

